# PNA-Based MicroRNA Detection Methodologies

**DOI:** 10.3390/molecules25061296

**Published:** 2020-03-12

**Authors:** Enrico Cadoni, Alex Manicardi, Annemieke Madder

**Affiliations:** Organic and Biomimetic Chemistry Research Group, Ghent University, Krijgslaan 281 S4, B-9000 Ghent, Belgium; Enrico.cadonI@ugent.be

**Keywords:** peptide nucleic acid (PNA), microRNA, fluorescence, templated reactions, nanoparticles, light-triggered, electrochemical biosensors, colorimetric detection

## Abstract

MicroRNAs (miRNAs or miRs) are small noncoding RNAs involved in the fine regulation of post-transcriptional processes in the cell. The physiological levels of these short (20–22-mer) oligonucleotides are important for the homeostasis of the organism, and therefore dysregulation can lead to the onset of cancer and other pathologies. Their importance as biomarkers is constantly growing and, in this context, detection methods based on the hybridization to peptide nucleic acids (PNAs) are gaining their place in the spotlight. After a brief overview of their biogenesis, this review will discuss the significance of targeting miR, providing a wide range of PNA-based approaches to detect them at biologically significant concentrations, based on electrochemical, fluorescence and colorimetric assays.

## 1. Introduction

### 1.1. MicroRNA Background and Importance as Biomarkers

After their first discovery in 1993 [1,2], microRNAs (miRNAs or miRs) have increasingly gained interest owing to their widespread role in controlling cellular functions. These short 20–22-mer RNA sequences are involved in the post-transcriptional fine regulation of multiple physiological processes of the cell, including proliferation, differentiation, cell death and signaling [3,4,5,6,7,8]. In Table 1, some of the major roles of the principal miRs are reported, including miRs which are dysregulated in specific pathologies.

**Figure 1:**
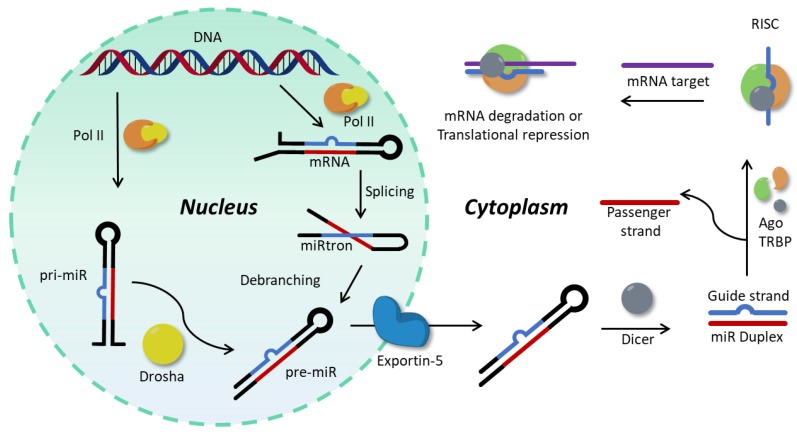
Schematic representation of the principal biogenetic pathways of microRNA (miR) formation and activity.

According to the canonical biogenesis, which is the principal biogenetic pathway [9], miR genes are transcribed by RNA Polymerase II into primary miRs (pri-miRs, see Figure 1). These hairpin structures are processed at the nuclear level into pre-miR by a microprocessor complex called Drosha, which cleaves the base of the hairpin [10,11] and exports the resulting pre-miRs to the cytosol via Exportin-5 [10,12]. Another, noncanonical, mechanism for production of pre-miR relies on the production of miR-introns (miRtrons), which are located in the intronic sequences of mRNA, and lead directly to pre-miRs during the splicing process of their host mRNAs, thus avoiding Drosha processing [13,14]. In the cytosol, pre-miRs are immediately processed by Dicer: the hairpin loop is cleaved, resulting in a mature duplex miR [15]. One of the two strands, called guide strand, associates with an Argonaut protein, forming the RNA-induced silencing complex (RISC) [9]. Recent investigations show that the Ago2 protein is loaded with the mature miR due to the action of the RISC-loading complex (RLC), composed by Ago2 itself, Dicer and the protein TRBP [16,17]. Both strands can be loaded into the RISC, but it has been described that the oligonucleotides with the lower thermodynamic stability at the 5′-end or those with an uracil in 5′ are preferentially loaded into the complex, while the other strand (passenger strand) undergoes degradation [18]. The RISC complex is then responsible for the post-transcriptional regulation of target mRNAs. This can happen via two principal mechanisms, i.e., direct mRNA degradation or translational repression. The stability of association between the guiding miR and the target mRNA determines the fate of the latter: moderate stability only results in target transcription block, while a strong association induces its degradation [17,19,20].

The key regulatory roles are dependent on the fine balance between the different components of the miR network, and, often, the alteration of the expression level of a single sequence can lead to the onset of diseases, including different forms of cancer as well as neurological pathologies [12,21,22,23]. Moreover, the total absence of miRs is associated with embryonic death, while a tissue-specific knockout of their biogenetic machinery leads to developmental defects [24]. 

This correlation between the dysregulation of specific miR sequences and the onset of specific pathologies has spurred the development of new methodologies for the precise quantification of these shorts sequences, with some of these methods already available on the market [25,26,27]. The use of DNA microarrays is a well-established method for low-cost high-throughput relative quantification. Such arrays are employed for a fast screening of up/down-regulation, but they show a low dynamic range and hybridization efficiencies largely depend on temperature, ionic strength and affinity between probes [28]. qRT-PCR is another well-established methodology that allows specific and sensitive detection of multiple miRs with precise quantification and is often used to validate microarray results on a specific set of targets. Finally, RNA sequencing is a high-profile methodology employed in the identification and quantification of miR sequences with high accuracy and selectivity in the discrimination of closely related sequences, but, on the other hand, relatively high amounts of sample are required and long analysis times as well as high costs limit its application.

### 1.2. Peptide Nucleic Acids

In the field of oligonucleotide targeting, peptide nucleic acids (PNAs) are emerging as a valid alternative to regular DNA probes for detection, because of their unique features. First reported in Copenhagen by Nielsen and coworkers in 1991, these DNA mimics have only recently started to become a widespread alternative for the use of its natural analogue [39]. The neutral poly-amidic backbone confers higher stability to the PNA:RNA duplex as compared to RNA:RNA or DNA:RNA complexes, with stabilities that are less affected by variations of the experimental conditions (i.e., ionic strength, solvent polarity or presence of chaotropic agents). At the same time, a stronger destabilization results from the presence of a mismatch in the duplex [40]. These features enable the employment of shorter sequences for the formation of a stable duplex with a target, and can be particularly useful in miR targeting, because of their short length and high homology in sequence, which in some cases may differ by only one base. Next to these advantages of PNA employment over DNA for miR detection, additionally, the non-natural backbone provides a unique stability towards nucleases and proteases, and a higher chemical stability compared to natural oligonucleotides, thus increasing the shelf-life of the probes [41]. As they are synthesized like a regular peptide, employing the well-established peptide solid phase synthesis, the introduction of modifications (such as small peptides sequences, ligands, fluorophores) directly on the solid support can be performed exploiting a broader range of chemistries as compared to the DNA case, where phosphoroamidite chemistry or in-solution couplings are required [42].

The use of PNA presents certain disadvantages that need to be taken into account during probe design. The neutral poly-amidic backbone, that ensures the formation of strong duplexes with natural nucleic acid targets, also leads to reduced solubility in aqueous media. This can be easily avoided by introducing charged and hydrophilic residues, such as charged amino acids (i.e., arginine, glutamic acid, etc.). This reduced solubility limitation is normally less important when operating at low micromolar concentrations normally employed in oligonucleotide detection, but it may still be a drawback when it comes to device fabrication. For example, the polyamidic backbone of PNA has shown to generate problems in the conjugation of thiolated probes to the surface of gold nanoparticles [43], and this requires the development of alternative protocols for gold decoration [44,45]. Additionally, the artificial nature of the PNAs prevents their recognition by enzymes, including those currently employed for amplification-based detection methodologies relying on DNA probes, such as ligases or polymerases.

Although these drawbacks may discourage their application, given their straightforward conjugation with peptide sequences and small functional groups and the aforementioned advantages in terms of duplex stability, PNAs have found numerous applications in nucleic acid targeting strategies and have been proposed as biomolecule-based drugs and as probes for diagnostic tools [46,47,48,49]. The aim of this review is to give an overview of the recent developments in PNA-based miR detection systems, with particular attention for the main advantages over regular DNA-based strategies.

## 2. Electrochemical Detection Methodologies

An electrochemical biosensor is, by definition, a device able to transduce a bio-molecular recognition event into a measurable electric signal, which can be amperometric, potentiometric or impedimetric [50,51]. A wide range of biomolecules can be employed as recognition element for the realization of this kind of device, ranging from antibodies and aptamers to cell receptors and lectins, and eventually allow for the detection of whole cells [52]. 

As recognition event, the hybridization of two complementary nucleic acid strands is particularly suitable for biosensing applications because of the high specificity of the base-pair [52]. Many electrochemical methodologies for DNA detection have been developed, providing in some cases effective and low-cost detectors without the need of expensive signal transduction equipment and, most importantly, enabling label-free detection [28,53]. 

Electrochemical miR biosensors constitute the main class of biosensors for miR detection. However, the high sequence homology and short length of miRs have remained difficult to overcome in the realization of reliable detection devices. In this field, PNA can help to overcome some of the specific miR-targeting problems. In addition, their use in electrochemical biosensors has shown to offer some intrinsic advantages over the use of standard DNA. Indeed, due to the neutral nature of the PNA backbone, most of the reported biosensors rely on the primary PNA–RNA hybridization event, which in turn induces the accumulation of negative charge on the sensor surface [54]. This charge accumulation can be used as signal per se, but, in order to enhance the sensitivity of the devices, additional reporter units, such as nanoparticles (gold, iron oxide, silver), metal complexes or organometallic compounds (such as ferrocene or ferricyanide), are often exploited [55,56].

### 2.1. Electrochemical Detection Methodologies Based on Hybridization

The simplest electrochemical detection platforms, as mentioned earlier, only rely on the charge accumulation obtained after RNA hybridization to the surface. In this case, the employment of PNA over regular DNA results in an immediate advantage. Given the charged nature of DNA, in fact, its use in these direct biosensing platforms often leads to high background noise, resulting in a lower sensitivity of the system.

**Figure 2:**
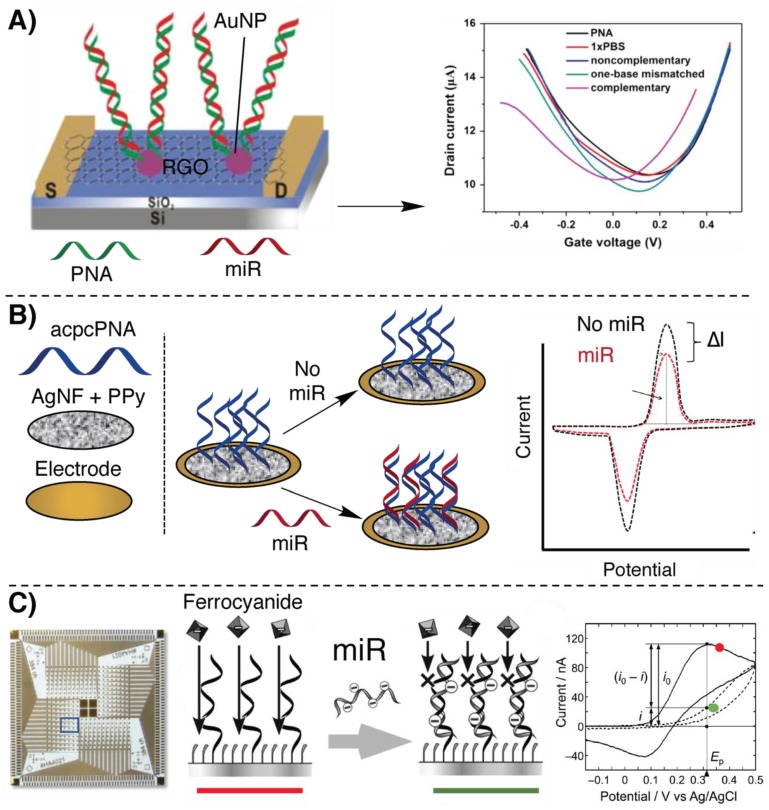
Illustration of some of the principal electrochemical methodologies based on hybridization for miR detection, relying on peptide nucleic acids (PNAs). (**A**) Field-effect transistor (FET)-based biosensor introduced in [59]. (**B**) Silver nanofoam (AgNF)-based biosensor decorated with acpcPNAs shown in [60]. (**C**) A 384-channel array on the Au/Cr surface based on the oxidation current of ferricyanide, described in [62].

In this context, Cai et al. reported the first example of a graphene oxide (GO) field-effect transistor (FET) biosensor for miR detection (Figure 2A). FETs are transistors consisting of a gate and two electrodes (source (S) and drain (D)) connected by a channel region typically made of a silicon-based material. Measurements with FET sensors rely on a change in channel conductivity. Control of the conductivity is possible through the application of a voltage between the gate and the source [57]. Based on a previously reported DNA-detection biosensor [58], a low-femtomolar detection of let-7b [59] could be realized. Reduced graphene oxide (RGO) was used as conducting material and deposited on the SiO_2_/Si surface of the FET. Gold nanoparticles (AuNPs) were used to decorate the RGO surface of the biosensor and thiolated PNA was then immobilized onto the AuNP surface. The role of the AuNPs was to increase the system sensitivity thanks to the high number of PNA probes that can be connected per NP. Upon hybridization of the miR with the PNA probes, the change in electrostatic potential, induced by charge accumulation at the biosensor SiO_2_/Si surface, results in a measurable V_GS_ change. Measurement of miR from serum was shown possible, allowing the detection of the presence of the target down to 1 fM.

Another example, where the charge accumulation is exploited for miR-21 detection, was recently reported by Kangkamano et al. [60]. This platform is based on silver nanostructures and relies on pyrrolidinyl PNA systems consisting of a D-prolyl-2-aminocyclopentanecarboxylic acid backbone (acpcPNA) developed in the Vilaivan group [61]. This sensor consists of a porous silver nanofoam (AgNF) coated with a thin layer of polypyrrole (PPy), obtained by electropolymerization. This conductive coating also provides the necessary amino groups for PNA anchoring. The foamy structure, together with the high density of amino functions, allowed the functionalization with a large amount of acpcPNA for miR capture, increasing the sensitivity of the system. The measurable signal of such a sensor was provided by observing the oxidation current of AgNF in phosphate buffer solution, employing cyclic voltammetry: hybridization with target miR increased the insulation of the surface, resulting in a decrease of such current, proportional to target concentration with a low limit of detection (LOD) of 0.2 fM (Figure 2B). 

Similarly, a 384-channel array was fabricated employing a photolithographically-produced Au/Cr electrode decorated with PNA probes (see Figure 2C) [62] for miR multiplex detection (miR-21, miR-17, miR-223), using relatively inexpensive and available materials. The Au/Cr surface of the electrode was functionalized with neutral PNAs, complementary to each miR target. Ferrocyanide was then used as electroactive species and its oxidation current was measured by the electrode. Recognition of the target miR results in a decrease of the oxidation current on the surface of each electrode, allowing the sensing of 384 possible targets with an LOD of 73.3 nM. Despite the rather low sensitivity, this methodology was presented as a cheap alternative for fast screening of PCR products.

In all the cases discussed above, the detection of the desired miR was only possible due to the negative charge accumulation at the biosensor’s interface. The relatively low LOD is here achieved without the use of signal amplification methodologies and was enabled by the characteristic, neutral PNA backbone. Similar approaches relying on DNA capturing probes are also possible, but in principle they exhibit lower sensitivity due to the presence of negative charges on the oligonucleotide backbone.

### 2.2. Nanopore-Based Methodologies

A relatively new entry in the field of oligonucleotide detection is the so-called nanopore. Providing a precise, label- and amplification-free quantification of single molecules presenting a charge, its use as detection platform is constantly growing and has found numerous applications in the analysis of oligonucleotides of interest, including miRs [63]. Nanopores are structures of nanometric size consisting either of a trans-membrane protein inserted into a phospholipidic membrane (“biological” nanopores) or a nanometric hole created in synthetic or semisynthetic materials (“solid-state” nanopores). Independently of their nature, the detection principle is based on the migration of the charged molecule through the pore, obtained by the application of a voltage across the membrane, and the monitoring of the current change [64]. In view of their neutral or positively charged nature, the application of PNAs as capturing probes represents once again the main advantage over the use of classical DNA probes.

**Figure 3:**
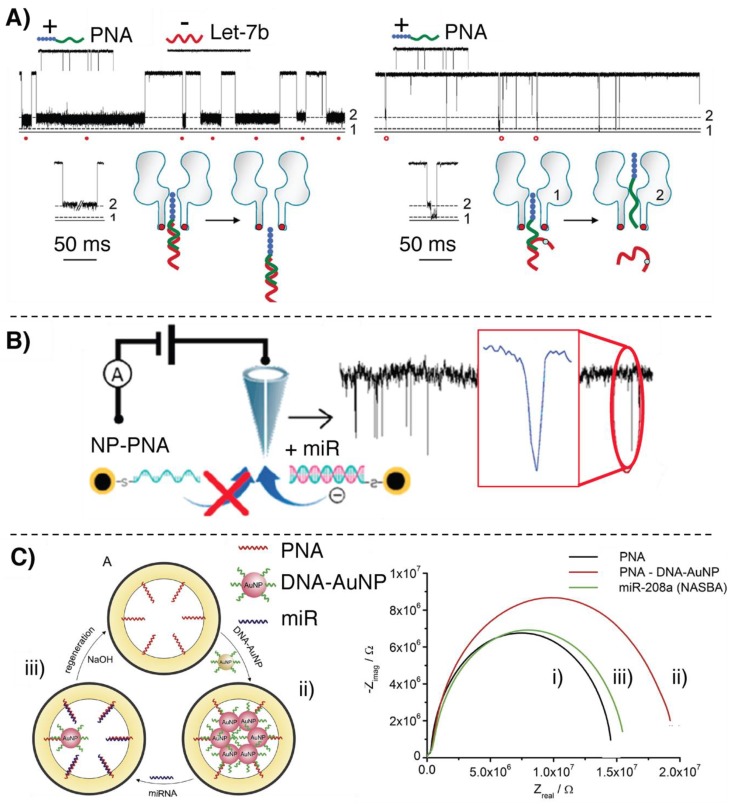
Illustration of the nanopore-based methodologies for miR detection relying on PNAs. (**A**) Nanopore sensing of let-7b, adapted with permission from [65]. Copyright 2013 American Chemical Society (**B**) Nanopore-based sensing of miR-21, adapted with permission from [66]. Copyright 2019 American Chemical Society (**C**) AuNP-displacement based biosensor described in [68].

The possibility to introduce positively charged amino acids in the PNA backbone was exploited in a work published by Tian et al., in which nanopore-based sensors were employed for selectively detecting let-7b [65]. Here, a polycationic peptide (TAT)-PNA carrier is exploited to form a supramolecular dipole with the target RNA sequence, leading to a channel blockade when a positive transmembrane potential is applied. At the same time the application of this potential allows repelling unrelated oligonucleotides from the pore, avoiding signal generation. In addition, the proposed system allows to easily discriminate the formation of mismatched complexes based on different channel blocking signatures. Detection of miR-7b with single-mismatch resolution proved possible (see Figure 3A).

An additional demonstration of the importance of the noncharged nature of the PNA backbone, enabling direct RNA detection without recurring to additional reporter groups, can be found in the work proposed by Wang et al., where nanopore sensing of miR-21 is enhanced by means of a core-shell iron-oxide–gold nanoparticle (Fe_3_O_4_–AuNP)-decorated PNA probe [66]. The positive potential applied to the nanopore attracts the negatively charged target and the passage through the pore of the RNA:PNA-AuNP hybrid enhances the amperometric signal generation as compared to standard miR detection. In addition, the magnetic core of the nanoparticle allows for isolation and quantification of the target from complex matrices (see Figure 3B). Similarly, gamma-PNA probes conjugated to polystyrene beads were also exploited by Zhang et al. to detect miR-204 and miR-210, with LODs of 1 and 10 fM, respectively [67]. 

Using solid-state nanopore arrays, Gyurcsányi’s group presented a biosensor based on the displacement of DNA–AuNPs conjugates for miR-208a detection in serum [68] (see Figure 3C). Gold nanopore arrays were functionalized with 18-mer PNAs complementary to the target miR. PNAs were then hybridized with 10-mer DNA-decorated AuNPs, designed to only weakly interact with the PNA probes on the nanopore. In this situation, the DNA–AuNPs block the ion current through the nanopore. In presence of the target miR, DNA displacement occurs accompanied by the release of the AuNPs, thus affording a measurable variation of system impedance. Based on the obtained results, the same group later reported on a new, optimized, nanopore array for potentiometric detection of the same miR, exploiting a different approach [69]. In this device, gold nanopores were decorated with positively charged PNAs to ensure anionic permselectivity, thus avoiding the passage of cations through the pore. Under these conditions, increase of KCl concentration results in a negative variation of the membrane potential, due to the Cl^−^ current. After hybridization with the negatively charged miR, the nanopore exhibits a cationic permselectivity, resulting in a positive membrane potential. It was demonstrated that this effect was correlated with miR concentration, showing an LOD of 100 pM.

### 2.3. Signal Amplification Methodologies

Examples of biosensors exploiting AuNPs were already reported above. In the aforementioned cases, the use of nanoparticles was justified as a means to increase the number of capturing probes on the biosensor surface (see [59]) or, in the case of nanopore-based detection, as a way to block the pore (see [68]). AuNPs, however, can additionally be employed in order to amplify the signal, enhancing the system sensitivity and generating lower background, thus increasing the LOD of the systems. For this purpose, various approaches may be used, including, for instance, the use of enzymes. The main advantage of signal amplification strategies resides in the possibility to facilitate the detection of low-abundant targets, particularly fitting miR detection in view of its low concentration in cell and serum [70].

**Figure 4:**
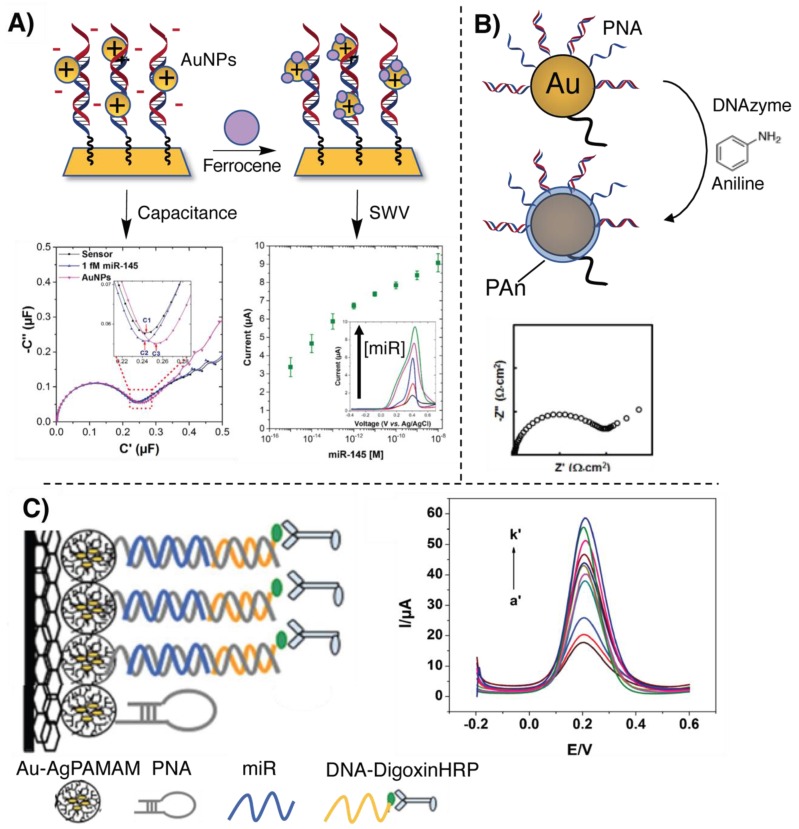
Illustration of the signal amplification and PNA-based electrochemical methodologies for miR detection. (**A**) Dual-mode detection of target miR-145, as presented in [71]. (**B**) Illustration of the miR-guided PAn biosensor proposed in [72] (**C**) Scheme of the biosensor described in [73].

An example of signal amplification for miR targeting is given by a work published by Jolly et al., in which the negative charge resulting from the hybridization with the target miR is used for the deposition of positively charged AuNPs. This PNA-based sensor able to detect miR-145 was realized employing a dual-mode detection methodology, allowing the sensing through the combination of an impedimetric and a voltammetric measurement in order to increase the sensitivity of the assay [71]. After hybridization of the negatively charged miR strand to the complementary PNA immobilized on a gold electrode, positively charged AuNPs were added to the sensor. The increase of capacitance due to the binding of the positive AuNPs to the negatively charged miR enables an initial impedimetric detection. To enable the complementary voltammetric detection, the AuNPs were decorated with a thiolated ferrocene as a redox marker for square wave voltammetry (SWV) (Figure 4A). The LOD of this system is 0.37 fM with a wide dynamic range, up to 100 nM.

Besides the use of nanoparticles, enzymatic amplification can also be used. Gao and coworkers developed a biosensor employing PNA for sub-femtomolar (0.5 fM) detection of let-7b [72]. A gold bead electrode was decorated with neutral PNA and subsequently hybridized with the target miR, resulting in a high negative charge density on the system. The electrode was then incubated in a cocktail of aniline, H_2_O_2_ and G-quadruplex-hemin DNAzyme which induces the polymerization of aniline on the electrode. In this case, the signal amplification is related to the controlled polymerization of polyaniline (PAn) on the sensor surface: the negative charge of the miR guides the PAn deposition through electrostatic interaction with the protonated aniline precursor. This thin polymeric layer on the electrode surface affects the electron transfer, thus permitting the read-out via electrochemical impedance spectroscopy (EIS) (see Figure 4B). Thanks to the neutral PNA backbone, instead of the anionic one of a DNA probe, aspecific adsorption of cationic aniline was minimized, resulting in a low background.

In the former cases, the role of a neutral PNA is crucial. The neutrality of the system is required in order to avoid the deposition of positive AuNPs or PAn in absence of the target miR, rendering the straightforward application of DNA capturing probes more difficult.

Alternatively, in the work of Xie and coworkers, the use of PNA instead of DNA is not dictated by the need for a neutral probe. They designed a biosensor for miR-126 employing a glassy carbon electrode modified with a chitosan–graphene composite and a polyamidoamine dendrimer composite containing gold and silver nanoclusters (Au-AgPAMAM), exploited for the functionalization with a PNA-hairpin capture probe, partially complementary to the target. After miR hybridization, the hairpin opens and allows the recognition of a digoxin-labelled DNA. Finally, the addition of a horseradish peroxidase anti-digoxin antibody conjugate allowed differential pulsed voltammetric detection (PVD), obtaining an LOD of 0.79 fM [73] (see Figure 4C). The advantage of using PNA, over regular DNA, is connected to the need for a stable hairpin with a short pairing region (a PNA hairpin with a 6-base pair stem region has a melting temperature higher than 37 °C, while a similar DNA hairpin would generally open up below 25 °C.

## 3. Fluorescence-Based Methodologies

Fluorescent probes able to induce a signal change after a molecular event (such as the formation of a duplex) are widely exploited for the realization of different applications. Enabling simple detection and high sensitivity, fluorescence can be used for monitoring target concentrations directly in biological samples as well as for in vivo imaging in living tissues [74]. One of the most widespread examples of fluorescence-based detection methodologies encompasses the so-called molecular beacon. These hairpin structures are labelled at the two edges with a fluorescent probe on one and a quencher on the other hand. Upon hybridization with the target, an increase in fluorescence signal is generated in response to a conformational change [75]. 

### 3.1. Fluorescence-Based Detection Methodologies Based on Hybridization

Fluorescent PNA probes are reported for a wide range of sensing applications [76]. Focusing on fluorescence-based methodologies developed for miR detection, there are several examples in literature where PNA beacons are used for in vivo imaging of miR. In this case, the advantage of employing PNA over DNA probes lies in the fact that that the hairpin structure requires a shorter stem region to be sufficiently stable, and this particularly fits the targeting of short sequences such as miRs. 

These applications are based on the internalization of the probe followed by simple hybridization with the target miR, which generates a turn on in the fluorescence readout. Different fluorophores are exploited for providing the signal, including fluorescein [77], cyanines [78] and chlorins [79]. 

On the other hand, graphene and graphene oxide (GO) [80] are often used in a dual role: as quencher as well as for cellular internalization [78,79,81,82]. These technologies provide additional examples in which the neutral backbone of PNA plays an important role and ensures strong π–π stacking interactions with GO with concomitant fluorophore quenching. Upon hybridization with the target RNA, the interactions weaken, provoking the release of the probe into the solution and the concomitant increase in fluorescence signal. In this context, the use of PNA rather than classical oligonucleotide probes is preferred in view of the negative charges of the GO at physiological pH that can cause electrostatic repulsion of negatively charged phosphate backbones, thus preventing the absorption of such systems and the fluorescence quenching effect.

**Figure 5:**
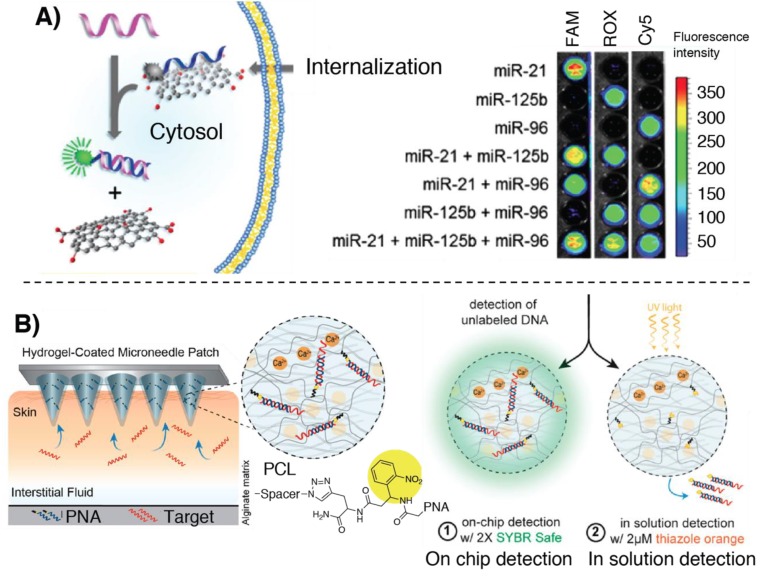
Illustration of some of the principal fluorescence-based methodologies for miR detection relying on PNAs hybridization. (**A**) Functioning of PNA–nano-graphene-oxide (PANGO) multiplexed analysis, adapted with permission from [82]. Copyright 2013 American Chemical Society. (**B**) Hydrogel-coated microneedle patch for sampling and detection of miR. Adapted with permission from [85]. Copyright 2019 American Chemical Society.

This effect was first demonstrated in the work published by Ryoo et al., providing the first example of a PNA–nano-graphene-oxide (PANGO) complex used for miR detection in living cells. Fluorescently labelled PNAs were quenched with NGO, due to the tight interaction between the probe and the nanosheets. Upon the addition of the target strands, the restored fluorescence provides the signal for quantification of miR. Recognition of the target miR thus provokes an increase in the fluorescence signal both in cell lysate as well as in the cytosol (Figure 5A). Exploitation of orthogonal fluorescent reporters was also demonstrated to be applicable to multiplex quantification of miR-21, miR-125b and miR-96 with LODs as low as 1 pM in solution or as little as 11.4 nM in living cells [82]. Later, Lee and coworkers reported on the application of a GO-quenched FAM-PNA system used for the detection of miR-193a. This was applied to a microfluidic culture system to track cellular differentiation via intercellular exosome delivery [81]. 

Nanoporous metal–organic frameworks (MOFs) exhibit quenching properties similar to graphene-based materials and can be exploited in similar applications for the detection of target oligonucleotides. As an example, Kang and coworkers proposed a UiO-66 nano-MOF based system coated with different fluorescently labelled PNA probes for the multiplexed detection of miR-21, miR-96 and miR-125b in cancer cells, with an LOD in solution of 10 pM [83].

The quenching properties of carbon nitride nanosheets (CNNS) were also exploited by Ju and colleagues, for the realization of a CNNS delivery system, coated with cyanine-labelled PNAs and a folic acid derivative, for the fluorescence detection of miR-18a in cancer cells, with a low background and high signal-to-noise ratio [84].

Ladame’s and Irvine’s groups recently developed a microneedle-based array skin patch for a selective and minimally invasive sampling technique that could, in principle, be applied for the isolation of miRs of interest from biological samples. For this purpose a microneedle array was coated with a PNA-modified alginate hydrogel, enabling the system to sample up to 6.5 µL in 2 min. The readout could be done either directly by dipping the microneedle in an intercalator dye solution, or by photocleaving the duplex first and adding the intercalator in solution [85] (see Figure 5B).

### 3.2. Templated Reactions

An alternative strategy to sense the presence of a target oligonucleotide strand is to use its sequence as a template for the formation of specific adducts [86]. Usually, two probes bearing complementary reactive functionalities are employed. Upon hybridization with the target strand, the reactive units are positioned in close proximity and reaction is promoted. The main advantage of such a system is that the effective concentration of the two reactive units is increased as a consequence of complex formation, making the reaction possible even at low concentrations. Furthermore, these methodologies infer a double selectivity to the system as both probes need to be hybridized at the same time with the target strand, thus allowing to avoid side reactions in presence of a mismatched target. On the other hand, in the case of templated ligations (i.e., covalent linkage between the two strands, as opposed to, e.g., label transfer reactions), the reaction product shows higher affinity for the template, preventing the possible recycling of the template [87]. In the past few years, several nucleic acid templated reactions have been developed with the aim of initiating a biological process, providing a specific signal for DNA/RNA detection, or synthesizing small molecules and macrocycles with affinity for the targeted structure. Many of these reactions, however, were performed using DNA as template, and not as many examples of templated strategies were used for miR sensing. The advantage of using PNA over regular DNA probes for templated reactions is to be found in the structural peculiarities of the target: given that miRs are short sequences, the use of two short DNA probes (10–11-mer) results in the formation of weaker (DNA)_2_:miR duplexes as compared to the stronger (PNA)_2_:miR ones. As a result, this lower stability translates to a higher chance that the required complex may be not formed, thus preventing the templated reaction. The most relevant PNA-based template methods are described in what follows.

#### 3.2.1. Hybridization-Triggered Templated Reactions

**Figure 6:**
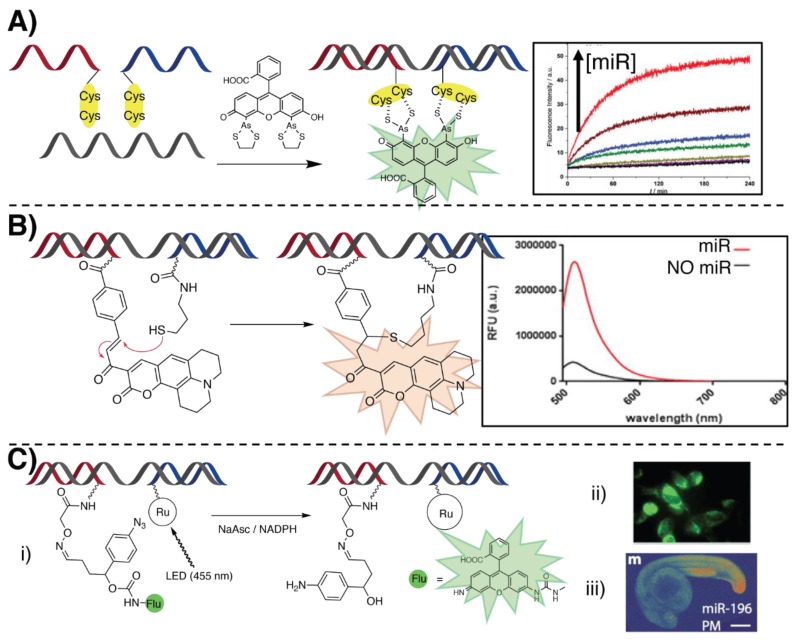
Illustration of some of the principal oligonucleotide templated reactions for miR detection relying on PNAs. (**A**) In situ fluorescence labelling of dicysteine PNAs for miR detection, through unmasking of FlAsh, described in [88] (**B**) Michael addition of a thiol-containing PNA to an α,β-unsaturated ketone of a nonfluorescent coumarin precursor. Adapted with permission from [90]. Copyright 2016 American Chemical Society. (**C**) Ruthenium (II)-based light-triggered reaction, freeing a quenched fluorophore upon light-mediated reduction of a pyridinium linker, as shown in [93]. (i) The reaction was conducted in cellulo (ii, [94]) and in vivo (iii, adapted with permission from [95]. Copyright 2016 American Chemical Society) for miR imaging.

In recent work by Seitz and co-workers, they reported on a methodology based on the miR-templated hybridization of two PNAs facing each other with a cys–cys dipeptide (see Figure 6A). Upon addition of a bi-arsenite fluorescent dye such as fluorescein arsenical hairpin binder (FlAsH, whose fluorescence is quenched when forming a complex with ethanedithiol), the arsenite binds the tetracysteine motif present in the template complex, releasing ethane-1,2-dithiol and uncaging the fluorophore [88]. They report an 80-fold increase of fluorescence when the two PNAs hybridize without gaps, which decreased by targeting an oligonucleotide with 1, 2 and 3 unpaired bases separating the targeted segments. By using RCA, they were able to reach a subnanomolar LOD for miR let-7a detection, with a signal 2.7-fold above the background at a concentration of 0.1 nM. The same typology of reaction was employed to label an anti-miR-17 PNA and for mRNA templated detection [89].

A templated Michael-addition was exploited by Ladame’s group in order to detect miR biomarkers in human serum for the diagnosis of prostate cancer (see Figure 6B) [90]. MiR-141 and 375 served as template to promote the formation of a fluorescent coumarin by reaction of a thiolated PNA with an α,β-unsaturated ketone of a nonfluorescent coumarin precursor present on the other strand. An optimal 3-oligonucleotide gap between the probe hybridization domains was maintained. The 1,4-addition resulted in a strong restoration of the fluorescence emission at 520 nm. The LOD of the system was estimated around 60 nM, maintaining a 5 µM stoichiometric concentration of the two PNA probes. A similar reaction was more recently exploited by the same group to detect miR-like DNA at concentrations as low as 100 pM in alginate-based hydrogel beads [91], delivering the first example of a templated reaction within a hydrogel. Recently, this reaction was also exploited in a lateral flow strip assay for the detection of miR-150 from plasma samples [92]. A biotinylated thiol-PNA (capture probe) was immobilized on the streptavidin-containing test line of the strip. The target miR was prehybridized with the coumarin-containing sensing probe and spotted on the loading pad of the strip. When eluted, the miR:coumarin-PNA complex was retained on the test line due to its hybridization with the capture probe. A fluorescence turn-on was then observed after drying the trip with a hair dryer. They report a final LOD of 9 nM, claiming to report the first example of an oligonucleotide-templated reaction on paper.

#### 3.2.2. Light-Triggered Templated Reactions

Quite often, the use of external stimuli, such as light, is exploited to gain additional spatiotemporal control over the activation of the system. Examples of these light-triggered templated reactions were developed in Winssinger’s group, in which they used ruthenium (II) chemistry to perform a photocatalyzed PNA ligation (see Figure 6C) [93]. In cellulo imaging of an miR-21 target strand was achieved by uncaging a protected fluorophore in a templated manner [94]. Light irradiation (455 nm) of a Ru(II) complex placed on a PNA strand triggered the reduction of an aromatic azide on the second PNA strand. The formation of the corresponding aniline induces a cascade reaction that leads to the uncaging of a rhodamine based pro-fluorophore. The reported reduction of the azide function was not achieved directly via interaction with the excited Ru(II) complex, but is reported to be mediated by the ascorbate, or by NADPH, naturally present in the cell. 

Later, the same reaction was employed for in vivo imaging of miR-9, 196 and 206 in a living vertebrate (zebrafish) [95], or for dsRNA-templated ligation [96], thanks to the formation of a dsRNA–PNA triplex with the final aim to target the pre-miR-31 hairpin, precursor of the mature miR-31. In this last report, the excitation of the Ru(II) catalyst induced the ascorbate-mediated reduction of an immolative pyridinium linker, with the final release of a difluoro coumarin leaving group. The ruthenium-containing probe does not form a ligation product with the facing strand, therefore permitting the recycling of the probe, which acts as a catalyzer for the reaction. The turnover frequency of the reaction was later on optimized with DNA analytes, reaching the impressive value of 102 h^−1^ and providing the fastest templated reaction reported to date [97].

### 3.3. Fluorescence-Based Detection Methodologies Featuring Signal Amplification

As described for electrochemical detection, signal amplification strategies can also be developed for fluorescence-based detection. In this section we describe examples of signal amplification methodologies applied in the context of miR detection, employing PNA probes.

**Figure 7:**
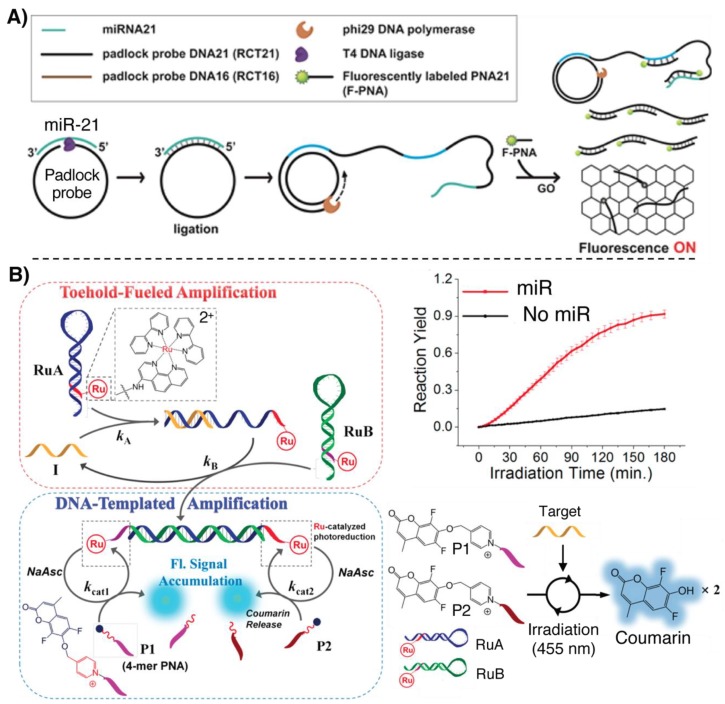
Illustration of some of the principal fluorescence-based methodologies for miR detection relying on PNAs, featuring a signal amplification strategy. (**A**) RCA-based detection of miR, adapted with permission from [98]. Copyright 2016 American Chemical Society. (**B**) Quadratic amplification applied for miR detection and release of coumarin, adapted with permission from [99]. Copyright 2019 American Chemical Society.

A first example involves the exploitation of rolling circle amplification (RCA), used in combination with fluorescently labeled PNAs for miR-21 detection [98]. The target miR acts as template to hold the two extremities of a DNA padlock probe in close proximity, allowing a T4 DNA ligase to circularize the probe. A polymerase is then able to use the circular DNA as template and the miR as primer to start the synthesis of the RCA product (RCAP). After this stage, GO and a FITC-PNA, of the same sequence as the target miR, are added, and if the RCAP is present it can sequester the PNA from the solution, preventing its quenching by the GO (Figure 7A).

In a recent work by Winssinger and colleagues, quadratic amplification is achieved by combining a DNA circuit based on two metastable DNA hairpins, decorated with a Ru(II) complex, with a light-triggered templated reaction for miR-21 detection [99]. When the target is present, it hybridizes with one of the hairpins of the circuit, allowing the formation of a long, single-stranded overhang. The single-stranded overhang is now able to hybridize with the second hairpin, displacing the target oligonucleotide, allowing the circuit to recycle the target and producing a dsDNA with two 4-mer overhangs. Two short 4-mer PNAs, bearing a pro-fluorescent coumarin linked with an immolative pyridinium spacer, hybridize to these two overhangs. Upon light irradiation at 455 nm, the Ru(II) photocatalyzed release of the coumarin results in an increased fluorescence signal which enables the detection of analyte down to 250 fM (Figure 7B). The methodology, here applied for sensing miR-21-like DNA sequences, can in principle be translated to miR detection. In this context, the use of PNA rather than DNA probes is crucial, given the short length of the overhangs which does not result in stable DNA:DNA duplexes.

## 4. Colorimetric Detection Methodologies 

Among other methodologies for sensing oligonucleotides, colorimetric assays have gained attention, providing a low-cost, effective and easy-to-handle alternative [100]. They are based upon a visual color change read-out that can be detected with bare eyes, without the need of expensive instruments. In many cases, this kind of assay relies on the use of gold or silver nanoparticles, which allow visual detection due to their fluorescence and luminescence properties, often combined with a high adsorption coefficient and an extended surface area for functionalization [101]. Despite the generally recognized advantages of such assays, including their cost-effectiveness, only few examples of colorimetric methodologies for miR sensing are available, and most of them are to be considered as “colorimetric assays” rather than proper biosensors [102].

### 4.1. Colorimetric Assays Based on Hybridization

**Figure 8:**
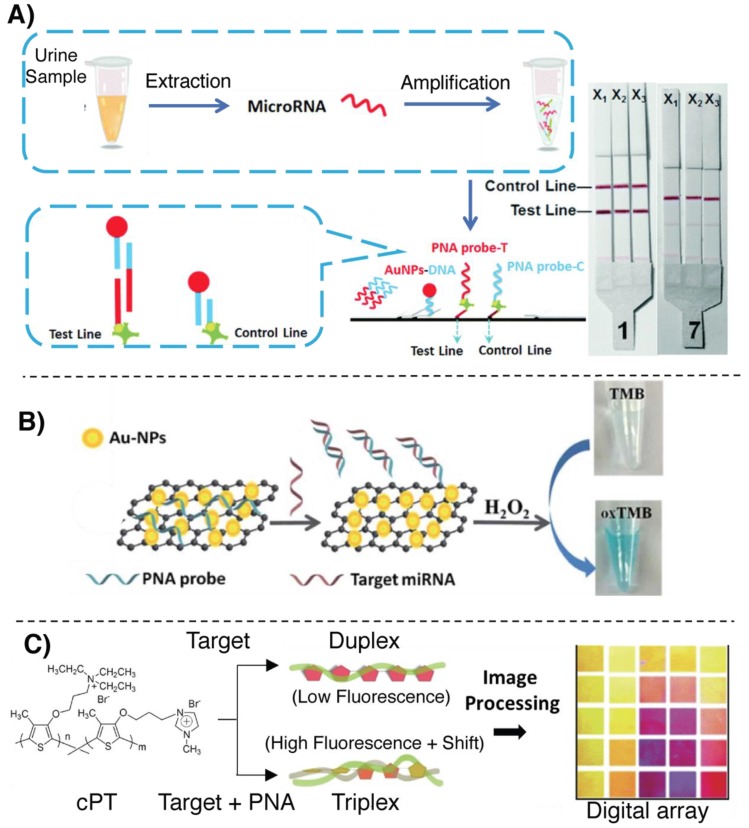
Illustration of some of the principal colorimetric methodologies for miR detection relying on PNAs. (**A**) Multiplexed, lateral flow strip detection of miR bladder cancer markers as illustrated in [103]. (**B**) Detection based on the peroxidase-like activity of graphene-AuNP nanohybrids shown in [107]. (**C**) Colorimetric/fluorescence-based detection of miR, relying on cPT:PNA:miR triplex formation, as described in [110].

Lateral flow strips (LFS), meet all the mentioned requirements, providing a simple, highly accessible and nearly-immediate read-out, without employing any additional instrument. As an interesting example of miR detection in LFS format, relying on the hybridization with a PNA probe, Cheng et al. designed a methodology for the detection of the bladder cancer markers miR-126, miR-182 and miR-152 from urine samples (see Figure 8A) [103]. As a means to enhance the identification precision and reduce detection costs, a multiplexed analysis of the miR markers was enabled by the realization of a trident-like LFS. This system allows a parallel singleplex analysis of the three markers, without lowering the sensitivity of the system as compared to the use of the same strip for multiple targets [104]. In a first step, target miRs are extracted from urine samples and amplified using a dual-isothermal cascade amplification based on base stacking hybridization and exponential isothermal amplification [105,106]. The resulting amplified mixture was loaded on the lateral flow together with a reporter AuNP–DNA conjugate and PNAs were only used in test- and control-lines to block and concentrate the amplicon:AuNP–DNA complex at specific position in the strip. A red coloration is observed as a consequence of the accumulation of AuNPs. The LOD reached with this biosensor is around 0.6 fM. 

Another example in which the interaction between PNA and graphene is exploited can be found in the work by Zhao et al. Here, the peroxidase-like catalytic activity of a graphene/AuNP hybrid was used for miR-21 detection (see Figure 8B) [107]. This system is able to induce the oxidation of 3,3’,5,5’-tetramethylbenzidine (TMB, a typical reagent exploited in ELISA tests) in presence of hydrogen peroxide. The catalytic activity of the system was passivated by the aspecific absorption of PNA probes on the surface and subsequently restored only in the presence of the target miR-21 sequence as a consequence of the detachment from the surface. As already mentioned in Section 3.1, the choice of using PNA is dictated by the need for a neutral molecule, in order to guarantee high adsorption of the probe to the graphene. The methodology showed an LOD of 3.2 nM and a low background.

Paper-based methodologies were also reported. An example is provided by Liedberg and coworkers, who developed a biosensing platform to sense the presence of miR-21. The proposed assay allows naked-eye detection of the target and does not require the use of any instrumentation with the exception of a UV lamp [108]. A polyvinylidene fluoride paper was soaked with positively charged poly(3-alkoxy-4-methylthiophene), a class of poly-thiophene (PT) polymers used as luminescent reporter. In absence of the target, the free PT maintains a nonplanar conformation, resulting in high fluorescence intensity (orange coloration) [109]. A neutral PNA complementary to the target was added to the system, and, upon further addition of complementary miR, the resulting formation of a PT:PNA:miR triplex retained the nonplanar conformation of the PT polymer, thus maintaining the orange-fluorescent coloration of the paper. In case of addition of noncomplementary miR, the interaction between the negative charges of the nucleic acids and the positively charged PT results in the formation of miR:PT planar adducts, quenching the PT fluorescence and causing a visible color-shift from orange to pink. Later, the same group reported the application of PT copolymers (cPT) for miR-21 detection in plasma, reporting an LOD of 10 nM without the use of expensive instrumentation for the read-out [110]. By controlling the monomer ratio, they were able to tune the inter-ring torsion of the co-polymer, obtaining a tunable colorimetric response upon interaction with miRNA with a better colorimetric response as compared to the PT predecessor (Figure 8C). More recently, using the approach described in [108], the same group designed a colorimetric array for targeting miR-21 directly from plasma. Without recurring to sample pretreatment or amplification, they were able to reach an LOD in the low nM regime (2 nM in plasma, 0.6 nM in distilled water) [111].

### 4.2. Colorimetric Assays Based on Templated Reactions

**Figure 9:**
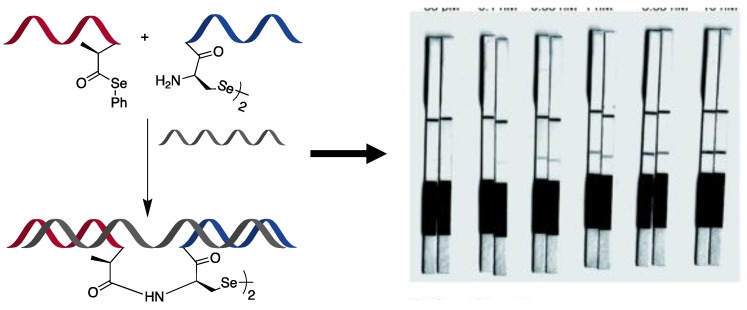
Illustration of the diselenide–selenoester ligation to a selenocysteine, templated by a target miR, used in a lateral flow strip assay as proposed in [112]. Published by the Royal Society of Chemistry.

Despite the aforementioned advantages in terms of immediacy of the outcome and low cost, there is only one recent example of a templated reaction applied for the realization of a colorimetric assay for miR detection. In this work by Winssinger’s group, a PNA–PNA templated ligation was developed for quick and selective detection of the target miR-31 [112]. Starting from a recently developed ligation chemistry, based on diselenide–selenoester ligation to selenocysteine [113], they developed an extremely fast RNA-templated reaction, providing a nearly immediate read-out (see Figure 9). The two PNAs were additionally labelled with fluorescein and biotin. When the two fragments hybridized on the target strand, the two reactive moieties were held in close proximity to react and provide a ligation product. The reaction mixture was then loaded on a lateral flow immunochromatographic strip, allowing the immobilization of the biotin-probe onto avidin present in the test zone of the strip. Finally, to sense the presence of the ligation product with the fluorescein-probe, anti-fluorescein antibody-coated AuNPs were employed, resulting in the formation of a band visible to the naked eye. This methodology provided an LOD of approximately 0.1 nM.

## 5. Other Methodologies

Finally, we here report on examples of methodologies described in literature in the past few years that do not fall within the main detection typologies reported above or that require the use of different detection techniques.

**Figure 10:**
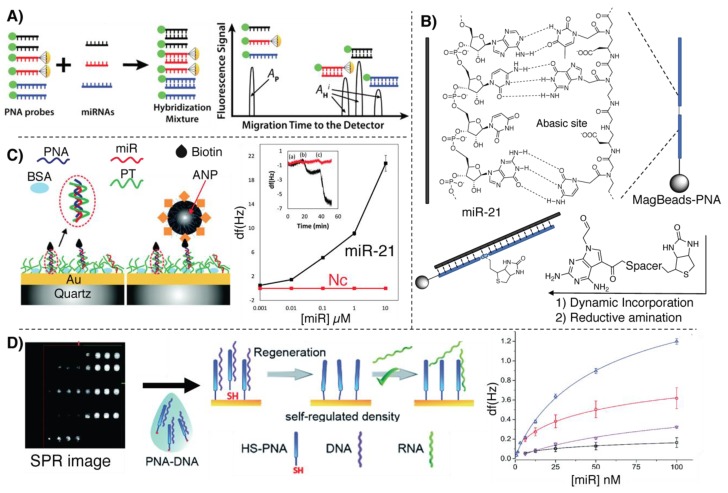
Illustration of other miR-detection assays based on PNAs. (**A**) DQAMmir analysis as adapted from [114]. Copyright 2016 American Chemical Society. (**B**) Bio-labeling of a PNA:miR-21 duplex with a biotinylated reactive base described in [115]. **(C**) Detection of miR-208a using the SPR-based methodology, adapted from [44]. Published by the Royal Society of Chemistry. (**D**) Illustration of the QCM-based methodology adapted from [116]. Published by the Royal Society of Chemistry.

An example can be found in the direct quantitative analysis of multiple miRs (DQAMmiR), a hybridization-based assay relying on capillary electrophoresis (CE), employing Alexa488-marked PNA probes (Figure 10A). The assay relies on the difference in charge between the neutral PNA and the negatively charged PNA–miR duplex, which are separable by CE in a 10 min window, allowing separation of multiple miRs by adding different peptide-tags on the PNA, thus changing the migration time to the fluorescence detector [114]. In this proof-of-principle study, Hu et al. managed to quantify, with high accuracy and precision, three different miRs simultaneously, based on the retention time of the complex and the signal areas. The LOD of such a system was reported to be 14 pM. 

Delgado-Gonzalez et al. recently proposed a detection system for single base resolution quantification of miR-21 levels in cell lysate, based on dynamic chemistry. By functionalization of magnetic nanospheres with a PNA containing an abasic site, they were able to induce a selective base coupling with a biotinylated reactive nucleobase (via reductive amination), templated by the target sequence [115]. In this particular case, as depicted in Figure 10B, this reductive amination is only possible in presence of the PNA backbone and cannot be applied to natural oligonucleotide probes. As additional advantage, the magnetic beads enable target pull-down from cell lysate prior to reaction with the labelled base. The fluorescence-based quantification was performed through biolabeling of the biotinylated probes with streptavidin–phycoerythrin or streptavidin–β-galactosidase conjugates. The LOD of the system was estimated to be around 35 pM.

An alternative application of the detection methodology proposed by Liedberg and coworkers, based on cationic PTs (please refer to Section 4.1), was applied for the detection of miR-21 on a quartz resonator surface [116]. In this work, gold-coated piezoelectric quartz crystals are functionalized with PT. The miR from the sample is then absorbed on the surface and a biotinylated PNA probe, complementary to the target, is allowed to hybridize with the target strand, yielding the formation of a PNA:miR:PT triplex structure, in a similar manner as described in [108]. The shift of resonance frequency and dissipation is then enhanced by the recognition of avidin-coated magnetite nanoparticles (ANP) (see Figure 10D). The LOD of the assay has demonstrated to be as low as 400 pM. 

Gyurcsányi and colleagues reported on a spotting methodology to efficiently immobilize PNAs on gold SPR chips, applied for the detection of miR-208a [44]. Given the tendency of PNA to aspecifically adsorb onto gold surfaces, and in order to achieve an ideal, controlled surface density, they prehybridized the thiolated PNAs with a complementary DNA strand (a DNA-miR-208a analogue) prior to surface functionalization. The DNA strand used for the prehybridization was then washed away with a NaOH solution, activating the surface to enable miR detection, which is performed monitoring the reflectance change of the surface (see Figure 10C). The system allows detection of miR-208a with a concentration as low as 140 fmol. 

## 6. Conclusions

The increasing importance of microRNA in early diagnosis of malignancies, neurodegenerative diseases and other pathologies is in line with the crescent understanding of all the biological processes regulated by these small oligonucleotides. Nowadays, the need for low-cost, efficient and high-throughput platforms to detect their presence at relevant biological concentrations is becoming more and more clear. In this review, we put emphasis on the use of PNA-based approaches, in view of their key advantages over regular oligonucleotides. These characteristics, combined with the possibility to adapt PNAs to all currently available detection methodologies, the wider accessibility of this synthetic oligonucleotide and the possibility for straightforward introduction of modifications, explain the occurrence of PNA as one of the main actors for the realization of efficient detection approaches. Although their use is associated with some drawbacks, we strongly believe that this is one of the cases in which the advantages outweigh the disadvantages, and we foresee an increased development of PNA-based biosensors and detection methodologies in the following years.

## Figures and Tables

**Table 1:** Overview of the principal miRs reported, summarizing the cellular and phenotypical effects of their upregulation or downregulation.

hsa-miR	Sequence (5′→3′)	Cellular Effect *	Phenotypical Effect	Ref.
**miR-15a**	UAGCAGCACAUAAUGGUUUGUG	Regulation of antiapoptotic *BCL2* gene (D)	Onset of chronic lymphocytic leukemia (CLL)	[29]
**miR-16**	UAGCAGCACGUAAAUAUUGGCG			
**miR-17**	CAAAGUGCUUACAGUGCAGGUAG	*E2F1* expression (U)	Cell proliferation	[5]
**miR-20**	UAAAGUGCUUAUAGUGCAGGUAG			
**miR-19a**	UGUGCAAAUCUAUGCAAAACUGA	upregulation of genes related to the immune response, T-cell activation, extracellular matrix and collagen network (U)	Regeneration of infarcted myocardium	[6]
**miR-19b**	UGUGCAAAUCCAUGCAAAACUGA			
**miR-21**	UAGCUUAUCAGACUGAUGUUGA	Antiapoptotic action via cell growth regulation. No effects on cell proliferation (U)	Glioblastoma, breast cancer onset	[30,31]
**miR-31**	UGCUAUGCCAACAUAUUGCCAU	Defects in protein p53 pathways (D)	Found in ovarian cancer	[32]
**miR-33**	GUGCAUUGUAGUUGCAUUGCA	Upregulated expression of cholesterol efflux transporters ABCA1 in liver (D)	Increased levels of HDL in plasma	[33]
**miR-138**	GCUACUUCACAACACCAGGGCC	Negative regulation of osteogenic differentiation of human mesenchymal cells (U)	Bone formation reduction	[8]
**miR-141**	UAACACUGUCUGGUAAAGAUGG	Upregulation of Androgen receptor transcriptional activity (U)	Prostate cancer onset	[34]
**miR-375**	UUUGUUCGUUCGGCUCGCGUGA			
**miR-145**	GUCCAGUUUUCCCAGGAAUCCCU	ARF6 overexpression (D)	Triple negative breast cancer onset	[35]
**miR-155**	UUAAUGCUAAUCGUGAUAGGGGUU	MYC overexpression (U)	CLL, Burkitt’s lymphoma, lung and colon cancer onset	[36]
**miR-210**	CUGUGCGUGUGACAGCGGCUGA	EFNA3 (VEGF signaling and angiogenesis) downregulation (U)	Upregulated in atherosclerotic plaques	[37]
**miR-221**	AGCUACAUUGUCUGCUGGGUUUC	KIT downregulation (U)	Modulation of erythropoiesis (CD34+)	[38]
**miR-222**	AGCUACAUCUGGCUACUGGGU			
**let-7**	miR family (miRs let-7a-2, let-7c and let-7g involved)	RAS protein upregulation (D)	lung cancer onset	[7]

* Upregulation = U; Downregulation = D.

## Search Strategy and Selection Criteria

The databases used to identify the most relevant papers include PubMed (https://www.ncbi.nlm.nih.gov/pubmed/), Web of Science (https://apps.webofknowledge.com/) and Scopus (https://www.scopus.com). The principal keywords used for identifying the papers here included were: miR, micro RNA, miRNA, microRNA, detection, assay, assays, PNA, PNAs, colorimetric, electrochemical, fluorescence, SPR, RNA, imaging, SPR, QCM. The keywords were used alone and/or in combination as follows: (miR OR microRNA OR miRNA OR micro RNA) AND (detection); (colorimetric OR electrochemical OR fluorescence) AND (detection OR assay) AND (miR OR microRNA OR miRNA OR micro RNA); (QCM OR SPR) AND (PNAs OR PNA) AND (miR OR microRNA OR miRNA OR micro RNA). Years included: 2013–2020.
